# Dosimetric comparison in sparing normal tissue dosage by using auto-SBRT planning in oligo liver tumors

**DOI:** 10.3389/fonc.2023.1273042

**Published:** 2023-11-09

**Authors:** Shu Zhang, Weiyi Zhan, Ni Zeng, Jiangping Yang, Maoqi Xiong, Wenjun Liao, Nianyong Chen, Jianghong Xiao

**Affiliations:** ^1^ Head and Neck Oncology Department, Cancer Center, West China Hospital, Sichuan University, Chengdu, China; ^2^ Department of Radiation Oncology, Cancer Center, West China Hospital, Sichuan University, Chengdu, China; ^3^ Cancer Center, West China Hospital, Sichuan University, Chengdu, China; ^4^ West China Clinical Skills Training Center, West China School of Medicine/West China Hospital, Sichuan University, Chengdu, China; ^5^ Department of Radiation Oncology, Radiation Oncology Key Laboratory of Sichuan Province, Sichuan Clinical Research Center for Cancer, Sichuan Cancer Hospital and Institute, Sichuan Cancer Center, Affiliated Cancer Hospital of University of Electronic Science and Technology of China, Chengdu, China; ^6^ Radiotherapy Physics and Technology Center, Cancer Center, West China Hospital, Sichuan University, Chengdu, China

**Keywords:** liver cancer, dose sparing, stereotactic body radiation therapy, volumetric-modulated arc therapy, auto-planning, number of half arcs

## Abstract

**Purpose:**

The study aimed to compare the dosimetric distribution of VMAT plans by increasing the number of half arcs in liver SBRT and investigate the effect by using automatic plan software in plan optimization.

**Method:**

Thirty-one patients with oligo liver tumors were randomly selected. VMAT treatment plans with different numbers of coplanar half arcs were generated.

**Result:**

Adding arcs significantly increased the PTV, *D_2%_*, *D_50%_*, and *CI*, but sacrificed the plan homogeneity. It also decreased the maximum dose of normal tissues such as the stomach, duodenum, and spinal cord and reduced *D_mean_*, *D_500cc_*, and *D_700cc_* for the liver. Nevertheless, the diminishing effect gradually decayed into three arcs. Meanwhile, the addition of arcs substantially extended the beam-on time.

**Conclusion:**

In the context of SBRT for oligo liver tumors, increasing the number of coplanar half arcs will improve PTV conformity and offer better protection for OARs, albeit at the expense of increased treatment duration. Considering the trade-off between plan quality and treatment efficiency, a three-arc plan may be more suitable for clinical implementation.

## 1 Introduction

Liver tumors are one of the leading causes of cancer-related mortality worldwide (1). Surgical resection with or without interventional therapy, target therapy, or systematic chemotherapy is the primary treatment option for hepatocellular carcinoma (HCC) or oligometastasis, but over 80% of patients present with unresectable tumors (2, 3). For those who are not operable, radiation therapy is another choice. However, radiation-induced liver disease (RILD) limits the use of radiotherapy in the long term.

Recently, the rapid development of radiotherapy techniques, such as intensity-modulated radiation therapy (IMRT) or volumetric-modulated arc therapy (VMAT), has expanded the indications of radiotherapy for liver tumors. Stereotactic body radiotherapy (SBRT) is an alternative option for early-stage HCC patients or oligometastases who are not eligible for surgery or interventional therapy, and it can also be used for locally advanced HCC (4, 5).

SBRT delivers high doses of radiation in a few fractions with better accuracy to the tumor target. Previous studies have shown that the success of SBRT is due to a higher biological effective dose (BED) and sparing of normal tissue (5). Current clinical investigations have unveiled SBRT’s effectiveness, manifesting in promising local control rates (1-year LC 56%–100%) and overall survival rates (1-year OS 32%–94%) (6–8), Additionally, SBRT has exhibited potential in slowing the progression of disease from an oligometastatic state to a polymetastatic one (9).

Different methods can be used for performing SBRT, including 3D-RT, IMRT, DCAT, VMAT, and Cyberknife. Among all these methods of delivery, VMAT is a type of rotational radiotherapy. VMAT is superior to other modalities due to its better dose distribution and treatment efficiency (10–15).

However, high-dose delivery to the target tissue can cause late effects, which can significantly damage nearby normal tissues (16–18). Therefore, it is crucial to reduce the dosage of the organs at risk (OARs) in SBRT treatment plans. It has been shown that the LC rate depends on the size and number of lesions, while the OS rate is strongly associated with liver function before treatment (19).

The quality of radiotherapy planning largely depends on the experience and skills of the medical physicists (20, 21). Manually setting and adjusting parameters is the mainstream in radiotherapy planning and optimization. It has been shown that optimization strategies greatly affect dose outcomes in liver SBRT plans (10). Therefore, we developed an automatic stereotactic body radiation therapy planning (ASP) program to improve the overall plan quality and consistency, prevent bias caused by different physicists while reducing the reliance on personal experience or skills, and accelerate the entire process. The ASP program has been previously evaluated in both the lung and liver with better reproducibility and repeatability (22–24). In this study, we aimed to investigate the efficacy of our ASP program in optimizing the liver SBRT plans by increasing the number of total half arcs in VMAT plans.

## 2 Materials and methods

### 2.1 Patient selection

Thirty-one patients with primary or metastatic liver tumors who underwent liver SBRT from 2017 to 2022 were randomly selected for analysis. The Clinical Research Committee of the study institute approved the protocol (Approval number 2022-1902). The need for written informed consent was waived by the Institutional Review Board.

### 2.2 Treatment plans

Patients were immobilized in a stereotactic body frame in a supine position with arms raised above the head. Portal venous phase contrast-enhanced computed tomography (CT) was obtained, covering the whole abdominal cavity. Diagnostic magnetic resonance imaging (MRI) was fused with multiple registration strategies to guarantee maximal accuracy and delineate the gross tumor volume (GTV). The clinical target volume (CTV) was coincident with GTV, and the planning target volume (PTV) was 5 mm axial and 10 mm cranio-caudal extension from CTV.

The ASP was used to design auxiliary structures, beams, initial objectives, and constraints. Then, the parameters were adjusted for optimization. The details of the ASP program were illustrated in our previous study (23). Objectives and constraints were further adjusted according to the prescription and limitations of OARs as recommended by Dr. Robert Timmerman (25). Parameter adjustment was based on each optimized objective value in the range of 10 to 30 times tolerance (tolerance = 0.0001). The total number of iterations per patient was arbitrarily set to 10. The minimum precision of the automatic adjustment was 2 cGy.

Four different VMAT plans were designed for each patient by adding the number of half arcs (181 degrees to 0 degrees); HA1 to HA4 stood for 1 to 4 half-arc VMAT plans, respectively. According to our previous study, the PTV dose was controlled in the range of 90%–110%, 90%–125%, and 90%–∞% of the prescription dose when utilizing the full arc. Heterogeneous PTV dosage improves lung SBRT planning (24). Considering the target volume located on the right side of the body, we used a half-arc and loosened the homogeneity limitation. The PTV dosage was controlled between 90% and 150%. Then, 48 Gy was delivered in four fractions for PTV; 99% of PTV received at least 90% of the prescription dose; and 95% of PTV was conformally covered by 100% of the prescription isodose curve. The heterogeneity index (*HI*) was calculated based on the International Commission on Radiation Units and Measurements report 83, and the conformity index (*CI*) was calculated according to the Paddick index (26). *D_2cm_* was the maximum dose (% prescription dose) to any point 2 cm away from the PTV in any direction; *R_50%_* was the ratio of 50% prescription isodose volume to PTV. Ten rings (each 5 mm in width) outside the PTV were considered to limit the OAR dose and evaluate the dose fall-off.

### 2.3 Plan analysis

Data are recorded as median value and their interquartile range (25%, 75%). The percentage differences were calculated as follows: (B − A)/A (A vs. B). The dose-volume parameters and delivery efficiency among the four plans were analyzed by the Friedman test. Values in different groups were compared by the Wilcoxon signed-rank test; *p* < 0.05 (two-tailed, Friedman) and *p* < 0.017 (α/3, two-tailed, Wilcoxon) were considered statistically significant.

## 3 Results

### 3.1 Clinical characteristics of included patients

The clinical characteristics of the 31 patients are summarized in **Table 1**. The average age of all the 31 patients was 55, ranging from 28 to 77. Seventeen (54.8%) patients were male, and 14 (45.2%) were female. Nine (29.0%) of them were diagnosed with primary HCC, and 22 (71.0%) were diagnosed with liver metastatic disease. Twenty-five (80.7%) patients were in the Child-Pugh A stage, only 1 (3.2%) patient was in the Child-Pugh B stage, and 5 (6.1%) of these patients remained uncategorized.

**Table 1:** Clinical characteristics of patients.

Variable	*N*	%
Age (years)		
Median (range)	55 (28-77)	
Gender		
Male	17	54.8
Female	14	45.2
Histology		
Primary	9	29.0
Metasis	22	71.0
Child-Pugh Stage		
A	25	80.7
B	1	3.2
NA	5	16.1

### 3.2 PTV dosage

The major parameters are summarized in **Table 2**. The average *D_2%_* of PTV was 62.23 Gy, 64.23 Gy, 64.63 Gy, and 64.56 Gy for HA1, HA2, HA3, and HA4, respectively. *D_2%_* in HA2 was significantly higher than in HA1 by 2.25% (*p* < 0.05), and there was no significant difference in the following plans. The average *D_50%_* of PTV in HA1 to HA4 was 56.64 Gy, 57.19 Gy, 57.13 Gy, and 56.68 Gy, respectively. *D_50%_* in HA2 was significantly higher than in HA1 by 0.52% (*p* = 0.004) (**Table 3**). In HA1 to HA4 plans, *D_98%_* ranged from 46.13 Gy to 46.19 Gy without significant differences (*p* = 0.357), and *D_99%_* ranged from 44.93 Gy to 44.99 Gy; however, there was no statistical difference (*p* = 0.318). Regarding the *CI*, HA2 and HA3 showed significantly higher *CI*, and the mean percentage differences were 0.59% (HA2 vs. HA1, *p* < 0.001) and 0.14% (HA3 vs. HA2, *p* = 0.003), respectively. However, the *HI* index was higher in HA2 than in HA1 (*p* < 0.001). There was no difference in HA3 or HA4. The average of MUs gradually increased from 2,021.9 to 2,430.4 (**Table 2**), and the mean percentage differences were 11.35% (HA2 vs. HA1, *p* < 0.001) and 2.31% (HA3 vs. HA2, *p* < 0.001). The median beam-on-time was 109, 128, 146, and 177 s for HA1, HA2, HA3, and HA4, respectively. The median beam-on-time significantly increased by 15.45% (HA2 vs. HA1, *p* < 0.001), 12.77% (HA3 vs. HA2, *p* < 0.001), and 17.61% (HA4 vs. HA3, *p* < 0.001), respectively.

**Table 2:** Plan parameter summary.

	HA1	HA2	HA3	HA4	p
	Median (25%, 75%)	Median (25%, 75%)	Median (25%, 75%)	Median (25%, 75%)	
PTV					
*D_2%_* (Gy)	62.23 (61.10, 62.80)	64.23 (62.47, 66.65)	64.63 (62.32, 65.93)	64.56 (62.31, 66.30)	0.005
*D_50%_* (Gy)	56.64 (56.14, 57.03)	57.19 (56.46, 58.39)	57.13 (56.20, 57.97)	56.87 (55.88, 57.78)	0.038
*D_98%_* (Gy)	46.13 (46.01, 46.24)	46.12 (45.88, 46.33)	46.19 (46.07, 46.31)	46.19 (45.97, 46.31)	0.357
*D_99%_* (Gy)	44.99 (44.63, 45.17)	44.97 (44.72, 45.24)	44.93 (44.73, 46.31)	44.99 (44.73, 45.24)	0.318
*HI*	0.28 (0.26, 0.30)	0.32 (0.29, 0.35)	0.32 (0.28, 0.35)	0.32 (0.28, 0.36)	0.003
*CI*	0.881 (0.864, 0.893)	0.885 (0.876, 0.904)	0.893 (0.877, 0.905)	0.891 (0.873, 0.905)	<0.001
MUs	2,021.9 (1,899.0, 2,197.9)	2,299.8 (2,142.0, 2,520.8)	2,386.4 (2,193.9, 2,540.5)	2,430.4 (2,177.9, 2,586.5)	<0.001
Spinal Cord					
*D_0.35cc_* (Gy)	6.23 (4.24, 7.91)	5.00 (3.77, 7.51)	4.82 (3.50, 7.14)	4.83 (3.78, 7.16)	0.001
*D_0.03cc_* (Gy)	7.16 (4.34, 8.41)	5.87 (4.02, 8.29)	5.49 (3.70, 7.75)	5.52 (4.12, 7.94)	0.002
Liver					
*D_500cc_* (Gy)	6.71 (2.95, 10.32)	6.50 (2.83, 9.99)	6.39 (2.84, 9.55)	6.29 (2.70, 9.48)	<0.001
*D_700cc_* (Gy)	1.94 (0.58, 3.72)	1.78 (0.57, 3.21)	1.61 (0.57, 2.78)	1.58 (0.55, 2.70)	<0.001
*D_mean_* (Gy)	9.46 (7.04, 11.08)	9.44 (6.88, 11.13)	9.27 (6.91, 11.05)	9.37 (6.81, 10.97)	<0.001
*V_5_* (%)	0.49 (0.37, 0.59)	0.48 (0.36, 0.59)	0.47 (0.37, 0.59)	0.47 (0.36, 0.58)	<0.001
Stomach					
*D_5cc_* (Gy)	5.69 (3.58, 10.58)	4.78 (3.03, 8.63)	4.32 (3.21, 8.50)	4.35 (2.88, 8.50)	<0.001
*D_0.03cc_* (Gy)	8.20 (4.36, 19.32)	7.54 (4.15, 16.16)	6.49 (4.00, 15.29)	6.56 (4.16, 14.63)	<0.001
Duodenum					
*D_mean_* (Gy)	0.87 (0.32, 2.38)	0.79 (0.16, 1.95)	0.76 (0.23, 2.03)	0.75 (0.22, 1.95)	<0.001
*D_5cc_* (Gy)	2.65 (0.55, 5.43)	1.98 (0.50, 4.66)	1.58 (0.50, 6.41)	1.62 (0.50, 4.74)	<0.001
*D_0.03cc_* (Gy)	5.71 (1.93, 13.96)	4.76 (10.67, 13.73)	4.44 (1.07, 14.53)	4.61 (1.13, 13.44)	<0.001
Kidney R					
*D_0.03cc_* (Gy)	13.97 (3.65, 22.01)	14.23 (2.77, 22.63)	13.27 (2.72, 22.71)	13.34 (2.70, 22.38)	0.612
*D_2cc_* (Gy)	7.80 (1.43, 17.02)	6.29 (0.95, 16.56)	7.31 (0.95, 16.90)	5.98 (0.89, 16.20)	0.717
*D_mean_* (Gy)	1.15 (0.27, 4.62)	0.84 (0.22, 4.44)	0.83 (0.23, 4.43)	0.87 (0.23, 4.50)	0.047
Bowel					
*D_mean_* (Gy)	0.13 (0.06, 0.38)	0.11 (0.05, 0.31)	0.11 (0.05, 0.33)	0.10 (0.05, 0.33)	<0.001
*D_2cc_* (Gy)	0.63 (0.28, 3.92)	0.60 (0.28, 2.80)	0.59 (0.26, 2.98)	0.59 (0.26, 2.85)	<0.001
*D_5cc_* (Gy)	0.56 (0.27, 3.28)	0.54 (0.24, 2.51)	0.52 (0.22, 2.76)	0.53 (0.22, 2.61)	<0.001
*D_0.03cc_* (Gy)	1.00 (0.34, 4.58)	0.81 (0.32, 3.52)	0.81 (0.32, 3.55)	0.81 (0.30, 3.55)	<0.001
Colon					
*D_mean_* (Gy)	0.48 (0.09, 1.04)	0.42 (0.08, 0.93)	0.41 (0.08, 0.92)	0.34 (0.07, 0.86)	<0.001
*D_2cc_* (Gy)	2.05 (0.44, 7.44)	1.75 (0.33, 7.98)	1.67 (0.43, 7.87)	1.46 (0.32, 7.90)	0.004
*D_20cc_* (Gy)	0.91 (0.20, 4.95)	0.89 (0.17, 4.17)	0.76 (0.18, 3.57)	0.85 (0.18, 3.77)	<0.001
*D_0.03cc_* (Gy)	3.86 (0.70, 10.14)	3.45 (0.49, 10.50)	3.81 (0.50, 10.46)	3.29 (0.42, 10.42)	0.014
Skin					
*D_mean_* (Gy)	0.58 (0.47, 0.97)	0.58 (0.46, 0.95)	0.57 (0.46, 0.95)	0.56 (0.45, 0.95)	<0.001
*D_10cc_* (Gy)	11.17 (9.63, 13.20)	11.29 (0.98, 13.21)	11.24 (9.77, 13.28)	11.28 (9.68, 13.28)	0.894
*D_0.03cc_* (Gy)	19.01 (14.72, 24.34)	17.98 (14.80, 23.88)	17.79 (14.70, 23.02)	18.07 (14.45, 23.42)	0.501

PTV, planning target volume; CI, conformity index; HI, homogeneity index; MU, monitor unit; D_Ncc_, minimum absorbed dose covering the N cc of the volume; D_V_, absorbed dose in fraction V of the volume; D_mean_, mean dose; V_D_, volume that receives at least the absorbed dose D Gy.

**Table 3:** Percentage difference in plan parameters across different plans.

	HA2 versus HA1		HA3 versus HA2		HA4 versus HA3	
	Median (25%, 75%)	*p*	Median (25%, 75%)	*p*	Median (25%, 75%)	*p*
PTV						
*D_2%_* (Gy)	−2.25% (−4.54%, −0.38%)	<0.001	0.07% (−1.06%, 1.29%)	0.185	−0.70% (−1.53%, 0.24%)	0.133
*D_50%_* (Gy)	−0.52% (−2.65%, 0.01%)	0.004	0.25% (−0.12%, 0.98%)	0.020	−0.16% (−0.70%, 0.44%)	0.813
*D_98%_* (Gy)	−0.05% (−0.41%, 0.22%)	0.164	−0.03% (−0.24%, 0.08%)	0.068	0.02% (−0.16%, 0.12%)	0.725
*D_99%_* (Gy)	−0.24% (−0.48%, 0.19%)	0.088	0.11% (−0.21%, 0.26%)	0.144	−0.11% (−0.27%, 0.20%)	0.504
*HI*	−7.38% (−13.93%, 0.60%)	<0.001	1.29% (−3.20%, 3.45%)	0.141	−2.37% (−4.75%, 1.53%)	0.123
*CI*	−0.59% (−1.07%, −0.15%)	<0.001	−0.14% (−0.48%, 0.06%)	0.003	−0.04% (−0.22%, 0.19%)	0.908
MUs	−11.35% (−13.93%, −8.06%)	<0.001	−2.31% (−4.94%, −0.42%)	<0.001	−1.03% (−3.67%, 1.21%)	0.113
Spinal Cord						
*D_0.35cc_* (Gy)	13.03% (1.99%,16.99%)	0.001	11.91% (4.75%, 25.53%)	0.232	−0.45% (−6.66%, 2.34%)	0.908
*D_0.03cc_* (Gy)	9.53% (1.05%, 18.21%)	0.004	0.83% (−4.07%, 7.90%)	0.247	−1.05% (−4.96%, 2.05%)	0.489
Liver						
*D_500cc_* (Gy)	1.96% (0.74%, 5.21%)	<0.001	1.36% (−0.61%, 3.92%)	0.011	0.70% (0.30%, 1.99%)	0.001
*D_700cc_* (Gy)	3.64% (0.31%, 13.43%)	<0.001	1.46% (−0.76%, 5.82%)	0.009	0.97% (−0.66%, 5.60%)	0.027
*D_mean_* (Gy)	0.88% (0.17%, 2.54%)	<0.001	0.37% (−0.14%, 1.69%)	0.001	0.18% (−0.25%, 0.81%)	0.091
*V_5_* (%)	2.10% (0.94%, 3.33%)	<0.001	0.66% (−0.57%, 2.67%)	0.005	0.35% (−0.13%, 1.06%)	0.015
Stomach						
*D_5cc_* (Gy)	12.86% (5.01%, 17.32%)	<0.001	1.51% (−0.01%, 8.61%)	0.003	−1.14% (−4.55%, 2.59%)	0.133
*D_0.03cc_* (Gy)	10.73% (3.60%,14.74%)	<0.001	4.59% (0.31%, 8.11%)	0.001	−1.22% (−5.06%, −0.34%)	0.075
Duodenum						
*D_mean_* (Gy)	7.80% (0.86%, 18.62%)	<0.001	1.04% (−1.64%, 3.10%)	0.051	1.30% (−1.81%, 2.62%)	0.223
*D_5cc_* (Gy)	3.64% (−0.20%, 21.19%)	0.001	0.17% (−1.33%, 2.43%)	0.261	0.98% (−2.06%, 2.57%)	0.144
*D_0.03cc_* (Gy)	4.30% (−0.41%, 18.68%)	0.001	0.40% (−1.43%, 4.20%)	0.100	0.54% (−3.94%, 3.02%)	0.232
Bowel						
*D_mean_* (Gy)	7.06% (0.00%, 13.62%)	0.008	0.96% (−4.27%, 4.73%)	0.261	2.89% (−0.13%, 7.76%)	0.009
*D_2cc_* (Gy)	5.44% (0.92%, 16.57%)	0.001	0.78% (−2.28%, 5.48%)	0.168	0.63% (−0.03%, 3.27%)	0.007
*D_5cc_* (Gy)	22.72% (14.91%, 42.50%)	0.003	0.78% (−2.19%, 5.33%)	0.191	1.33% (−0.25%, 4.87%)	0.015
*D_0.03cc_* (Gy)	5.03% (0.19%, 14.53%)	0.008	0.86% (−2.24%, 4.13%)	0.181	0.40% (−1.11%, 2.43%)	0.061
Colon						
*D_mean_* (Gy)	3.87% (−1.08%, 15.50%)	0.003	1.55% (−0.90%, 5.20%)	0.011	1.42% (−0.69%, 7.36%)	0.077
*D_2cc_* (Gy)	2.74% (−1.15%, 7.68%)	0.041	1.90% (−0.96%, 3.56%)	0.048	−0.13% (−1.30%, 4.53%)	0.223
*D_20cc_* (Gy)	5.19% (1.00%, 14.85%)	<0.001	2.91% (−3.23%, 7.77%)	0.015	1.92% (−2.37%, 5.97%)	0.237
*D_0.03cc_* (Gy)	2.57% (−2.40%, 9.73%)	0.049	0.88% (−2.14%, 3.84%)	0.097	0.13% (−0.90%, 4.00%)	0.141
Kidney R						
*D_0.03cc_* (Gy)	−0.37% (−2.10%, 4.169%)	0.328	1.13% (−3.31%, 0.58%)	0.019	1.24% (−0.59%, 2.63%)	0.019
*D_2cc_* (Gy)	1.98% (−1.05%, 14.25%)	0.013	0.51% (−1.28%, 1.96%)	0.161	0.23% (−1.48%, 1.25%)	0.080
*D_mean_ (Gy)*	4.46% (−0.67%, 14.10%)	0.004	0.43% (−0.94%, 4.97%)	0.075	0.39% (−1.81%, 2.76%)	0.059
Skin						
*D_mean_* (Gy)	1.50% (−0.099%, 3.77%)	<0.001	0.51% (−0.15%, 1.33%)	0.003	0.32% (−0.36%, 0.68%)	0.061
*D_10cc_* (Gy)	0.21% (−1.14%, 1.11%)	0.303	0.24% (−0.42%, 0.78%)	0.160	−0.28% (−1.39%, 0.25%)	0.059
*D_0.03cc_* (Gy)	−0.42% (−0.30%, 0.08%)	0.051	0.21% (−1.15%, 1.50%)	0.267	−1.00% (−2.40%, 0.25%)	0.055

Percentage differences were calculated as (B − A)/A (A vs. B).

PTV, planning target volume; CI, conformity index; HI, homogeneity index; MU, monitor unit; D_Ncc_, minimum absorbed dose covering the N cc of the volume; D_V_, absorbed dose covering a specified fractional volume V; D_mean_, mean dose; V_D_, volume receiving at least the absorbed dose D Gy.

### 3.3 OAR dosage

All the parameters of OARs are summarized in **Tables 2**, **3**.

#### 3.3.1 Spinal cord

When evaluating the spinal cord damage, the *D_0.35cc_* and *D_0.03cc_* were gradually decreased from HA1 to HA4. The maximum dosage, including *D_0.35cc_* and *D_0.03cc_* of HA2, was decreased by 13.03% (CI: 1.99% to 16.99%, *p* = 0.001) and 9.53% (CI: 1.05% to 18.21%, *p* = 0.003) compared to HA1, respectively.

#### 3.3.2 Duodenum and bowel

When considering duodenum damage during liver SBRT, *D_5cc_* and *D_0.03cc_* were the most commonly used parameters. *D_5cc_* and *D_0.03cc_* were significantly reduced by 3.64% and 4.30% from HA1 to HA2, respectively. However, increasing more arcs did not decrease the *D_0.03cc_*. Meanwhile, the *D_mean_* of duodenum was lower in HA2 than in HA1 (*p* < 0.001). *D_2cc_*, *D_5cc_*, and *D_0.03cc_* were used for the bowel, which includes all segments of the small intestines except the duodenum. From HA1 to HA2, *D_2cc_*, *D_5cc_*, and *D_0.03cc_* significantly decreased by 5.44%, 22.72%, and 5.03%, respectively.

#### 3.3.3 Right kidney

The right kidney is adjacent to the lower segment of the liver; thus, its safety is critical. Compared to HA1, *D_2cc_* decreased by 1.98% in HA2 (*p* = 0.013); however, *D_0.03cc_* was not significantly different. *D_mean_* for the right kidney also decreased by 4.46% from HA1 to HA2 (*p* = 0.003). No significant improvement was observed in HA3 or HA4.

#### 3.3.4 Stomach

For the stomach, from HA1 to HA4, the median *D_5cc_* was 5.69 Gy, 4.78 Gy, 4.32 Gy, and 4.35 Gy, and the median *D_0.03cc_* was 8.20 Gy, 7.54 Gy, 6.49 Gy, and 6.56 Gy, respectively. From HA1 to HA2, *D_5cc_* and *D_0.03cc_* reduced 10.73% and 12.86%, respectively.

#### 3.3.5 Normal liver

For normal liver, *D_500cc_, D_700cc_*, *D_mean_*, and *V_5%_* were evaluated. *D_500cc_, D_700cc_*, *D_mean_*,and *V_5%_* were all lower in HA2 than in HA1 and improved by 1.96%, 3.64%, 0.88%, and 2.10%, respectively (HA2 vs. HA1). Compared with HA2, *D_500cc_, D_700cc_*, *D_mean_*, and *V_5%_* improved by 1.36%, 1.46%, 0.37%, and 0.66% in HA3, respectively. Thus, adding arcs may spare the normal liver.

### 3.4 Dose fall-off

**Figure 1:**
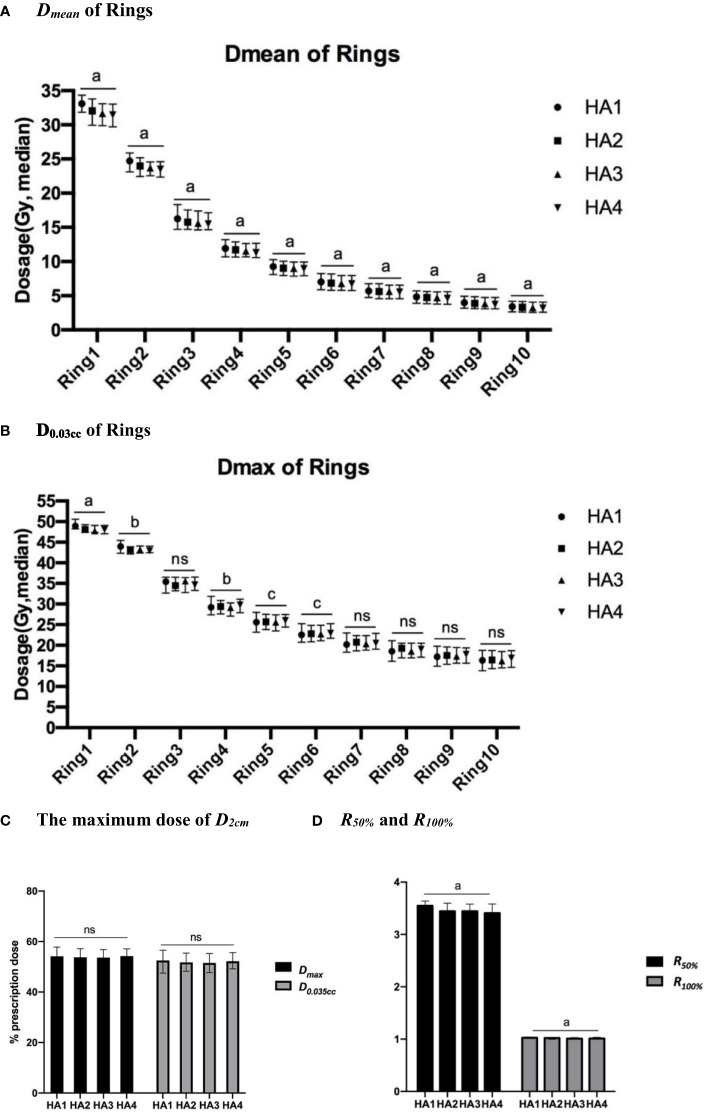
Dose fall-off analysis of different SBRT plans. Dose fall-off analysis among four stereotactic body radiation therapy (SBRT) treatment plans: **(A)** *D_mean_* (median value) of 10 rings; **(B)** *D_0.03cc_* (median value) of 10 rings; **(C)** The maximum dose (median) of *D_2cm_*, **(D)** The *R_50%_* and *R_100%_* of all patients. *a*, p<0.0001; b, p<0.01; c, p<0.05; ns, p≥0.05.

The dose fall-off curves based on *D_mean_* and *D_0.03cc_* of PTV are presented in **Figures 1A, B**, and the differences are shown in **Table 4**. *D_mean_* dramatically decreased from ring 1 to ring 4, and then moderately decreased from ring 5 to ring 10. Using two arcs decreased the *D_mean_* of each ring compared with using one arc. The value of *D_mean_* got smaller with increasing arcs. However, there was no statistical difference between HA4 and HA3 as shown in **Table 4**. The *D_0.03cc_* in ring 1, ring 2, ring 4, and ring 6 was significantly lower in HA2 than those in HA1.

**Table 4:** Percentage difference in plan parameters in different plans.

	HA2 versus HA1		HA3 versus HA2		HA4 versus HA3	
	Median (25%, 75%)	*p*	Median (25%, 75%)	*p*	Median (25%, 75%)	*p*
D_*mean*_						
Ring 1 (Gy)	2.69% (0.96%, 5.55%)	<0.001	0.36% (−0.16%, 1.84%)	0.033	0.29% (−0.36%, 1.66%)	0.227
Ring 2 (Gy)	2.24% (0.87%, 4.73%)	<0.001	0.27% (−0.14%, 1.61%)	0.021	0.39% (−0.30%, 0.97%)	0.144
Ring 3 (Gy)	1.90% (0.34%, 4.56%)	<0.001	0.36% (0.02%, 1.37%)	0.004	0.36% (−0.26%, 0.81%)	0.133
Ring 4 (Gy)	1.86% (0.17%, 3.92%)	<0.001	0.37% (0.01%, 1.22%)	0.003	0.13% (−0.15%, 0.73%)	0.149
Ring 5 (Gy)	1.59% (0.46%, 3.74%)	<0.001	0.45% (−0.03%, 1.20%)	0.003	0.13% (−0.16%, 0.76%)	0.189
Ring 6 (Gy)	1.44% (0.04%, 3.78%)	<0.001	0.44% (0.01%, 1.29%)	0.003	0.27% (−0.15%, 0.78%)	0.095
Ring 7 (Gy)	1.43% (−0.04%, 3.66%)	<0.001	0.47% (0.06%, 1.42%)	0.003	0.18% (−0.15%, 0.74%)	0.095
Ring 8 (Gy)	1.53% (0.04%, 3.73%)	<0.001	0.60% (0.22%, 1.54%)	0.001	0.19% (−0.16%, 0.72%)	0.149
Ring 9 (Gy)	1.44% (0.31%, 3.85%)	<0.001	0.61% (0.23%, 1.70%)	0.001	0.20% (−0.13%, 0.79%)	0.156
Ring 10 (Gy)	1.47% (0.47%, 3.63%)	<0.001	0.79% (0.08%, 1.84%)	0.001	0.31% (−0.23%, 0.86%)	0.235
D_*0.03cc*_						
Ring 1 (Gy)	2.34% (−0.08%, 2.94%)	0.001	0.12% (−0.90%, 1.15%)	0.532	0.18% (−0.81%, 0.92%)	1.005
Ring 2 (Gy)	2.06% (−0.36%, 4.79%)	0.005	0.01% (−0.72%, 0.68%)	1.251	0.34% (−0.41%, 0.67%)	0.331
Ring 3 (Gy)	1.21% (−1.02%, 3.17%)	0.087	−0.10% (−1.39%, 1.15%)	1.025	−0.08% (−1.11%, 0.58%)	0.641
Ring 4 (Gy)	1.86% (0.48%, 3.43%)	0.003	−0.21% (−1.47%, 1.10%)	0.965	−0.60% (−1.76%, 0.53%)	0.113
Ring 5 (Gy)	0.47% (−1.74%, 3.09%)	0.227	0.25% (−0.99%, 1.14%)	0.947	−1.09% (−2.44%, −0.06%)	0.001
Ring 6 (Gy)	0.67% (−0.65%, 3.68%)	0.045	−0.10% (−0.98%, 1.47%)	1.127	−1.09% (−2.07%, 0.37%)	0.005
Ring 7 (Gy)	0.86% (−1.63%, 2.76%)	0.352	−0.17% (−1.59%, 1.77%)	1.167	−0.34% (−2.27%, 1.14%)	0.309
Ring 8 (Gy)	0.53% (−2.73%, 2.40%)	0.927	0.44% (−1.33%, 1.65%)	0.947	−0.70% (−1.98%, 0.80%)	0.227
Ring 9 (Gy)	0.61% (−3.36%, 2.61%)	1.208	0.60% (−1.07%, 1.26%)	0.673	−0.20% (−1.91%, 1.77%)	0.624
Ring 10 (Gy)	0.12% (−4.12%, 2.88%)	1.005	0.47% (−1.79%, 2.26%)	0.760	−0.20% (−2.00%, 2.07%)	1.085
*R_50%_*	1.67% (0.40%, 3.08%)	0.001	0.18% (−0.37%, 0.84%)	0.289	−0.19% (−0.58%, 0.65%)	0.927
*R_100%_*	0.54% (0.15%, 1.07%)	<0.001	0.14% (−0.06%, 0.44%)	0.016	0.00% (−0.19%, 0.22%)	0.985

Percentage differences were calculated as (B − A)/A (A vs. B).

D_*mean*_, mean dose; V_D_, volume receiving at least the absorbed dose D Gy; R_50%_, the ratio of 50% prescription isodose volume to the PTV volume; R_100%_, the ratio of prescription isodose volume to the PTV volume.

In this study, we used *D_2cm_*, *R_50%_*, and *R_100%_* to evaluate dose spillage. From HA1 to HA4, the *D_max_* and *D_0.035cc_* of *D_2cm_* (**Figure 1C**) ranged from 53% to 54% and from 52% to 53%, respectively. *D_max_* and *D_0.035cc_* showed no significant difference after increasing the number of arcs. From HA1 to HA4, the value of *R_50%_* was 2.61 to 5.63 in all patients. The mean value of *R_50%_* significantly decreased by 1.67% in HA2 compared to HA1. *R_100%_* ranged from 0.98 to 1.20 in all the plans. *R_100%_* was lower in HA2 than in HA1 and decreased by 0.14% in HA3 compared with HA2 (**Figure 1D** and **Table 4**).

## 4 Discussion

To our knowledge, this is the first study to use the self-coding automatic software program to generate the liver SBRT plan and compare the efficacy by adding the number of half arcs in the VMAT plans. This system was previously validated in lung tumors (22, 24), and this ASP for each plan can be similarly optimized. Thus, we can minimize the impact of the experience or clinical preference of different physicists and reduce the deviations caused by manual adjustments.

In the past few years, IMRT has been the most commonly used method to generate SBRT plans (27). Recently, VMAT became popular due to its more flexible gantry rotation, variable dose rate, and dynamic multileaf collimator movement (28). Thus, VMAT can improve the dose distribution and shorten the treatment duration in both conventional and SBRT radiotherapy plans (29). Recent studies showed that a partial-arc SBRT plan can better protect normal tissues without sacrificing the target dose in lung SBRT (30). Considering that the anatomical location of the liver tumor is mostly unilateral, we set up the half-arc rotating to cover the whole liver instead of using a full arc. Then, we used *D_2cm_*, *R_50%_*, *CI*, and *HI* index to analyze the dosimetric characteristics. *D_2cm_* and *R_50%_* were normally used to evaluate the low-dose spillage in the SBRT plan, according to RTOG0813 (31). In the standard criteria, the maximum dose of *D_2cm_* should be limited to 50%–77% of the prescription dose, and the *R_50%_* value should be stipulated to <2.9–5.9. In our plans, the *D_0.03cc_* of *D_2cm_* was approximately 53%–54% due to the relatively larger size of liver tumors, which was acceptable. Seven of the thirty-one patients did not meet the *R_50%_* criteria in HA1. Using multiple arcs helped four of the seven the suggested goal in HA2 to HA4. On the other hand, as the number of arcs increased (from one to three arcs), the *CI* of PTV improved, indicating that increasing arcs enhanced conformity. However, the four-arc plan did not improve the *CI*. Thus, more flexible dose delivery in the VMAT plan can limit dose spillage without compromising conformity.

Multiple concentric rings outside the PTV were used for dose fall-off limitations, which have been studied among stereotactic radiosurgery plans (32, 33). In this study, we conducted 10 concentric rings evaluating dose fall-off and measured the dosimetric parameters for each ring. Analyzing the *D_0.03cc_* of rings indicated that using two half-arcs enhanced normal tissue safety. However, no further progress was observed in the HA3 or HA4 optimization plans. Moreover, the dose fall-off curve based on the *D_mean_* of rings was steep from ring 1 to 4 and then became mild from ring 4 to 10. Previous studies have demonstrated the advantages of the SBRT plan with heterogeneous plan optimization (24). Thus, we loosened the limitations of *HI* when establishing the optimization characteristics, providing more conformal radiation plans. In previous studies, multiple arcs increased beam-on-time (34, 35). In this study, the longer beam-on time was observed after using more arcs.

The most dangerous toxicity in liver SBRT is RILD. As a classic parallel organ, the risk of RILD depends on the dose of irradiation and the volume of the irradiated organ (18). Several studies have established different models of dose limitation to minimize the risk of RILD. A group initially used the dose constraint of at least 700 cc of normal liver <15 Gy in three fractions, and no patient experienced grade 3 liver or intestinal dysfunction (36). This clinical trial included patients with liver metastases; however, patients with primary HCC are more susceptible to RILD due to hepatic dysfunction or cirrhosis. Some studies suggested that for patients in the Child-Pugh B stage, the dose to one-third of the uninvolved liver was restricted to ≳18 Gy, and the dose to ≧500 cc of the uninvolved liver was restricted to <12 Gy in five fractions (37, 38).

Meanwhile, the mean dose of a normal liver is also required, according to the QUANTEC report in 2010. The mean dose should be <15 Gy for liver metastases in three fractions and <20 Gy for liver metastases in six fractions (18). In our study, the mean dose of 700 cc of the normal liver and the mean dose of the normal liver were below the constraints. *D_500cc_* ranged from 6.71 Gy to 6.29 Gy which met the limitations mentioned above. *D_mean_* ranged from 9.27 Gy to 9.33 Gy. Increasing the number of arcs (one to four arcs) decreased *D_500cc_*, *D_700cc_*, and *D_mean_*, resulting in a lower probability of RILD.

The right side of the kidney is anatomically adjacent to the liver. Previous studies showed that, as a mixed serial and parallel organ, the kidney obeys a volume–dose-response relationship. Cassady found that a total dose was associated with a 5% and 50% risk of injury after 5 years of 18–23 Gy and 28 Gy radiotherapy, respectively (39). Nevertheless, Cheng et al. showed that a dose of 9.8 Gy was associated with a 5% risk of kidney toxicity (40). In 2010, the AAPM published a recommendation for kidney dose limitation. Based on the threshold dose, 200 cc of the kidney volume should be exposed to less than 16 Gy in three fractions or 17.5 Gy in five fractions (41). Based on RTOG1112, the mean dose of bilateral kidneys should be less than 10 Gy. In this study, the mean dose of the right kidney ranged from 0.83 Gy to 1.15 Gy in HA1 to HA4. Among the 31 patients, only one patient had a *D_mean_* of 4.9 Gy. From HA1 to HA4, *D_0.03cc_* ranged from 13.27 Gy to 14.23 Gy, and *D_2cc_* ranged from 5.98 Gy to 7.80 Gy. *D_mean_* reduced by 4.46% and 0.43% from HA1 to HA2 and from HA2 to HA3, respectively. Using a multiple-arc plan may help reduce the dose to the kidney (HA1 to HA4) when *D_0.03cc_* is significantly decreased.

Among the serial organs, the spinal cord, duodenum, colon, and stomach were the most critical organs. Kopek et al. found that the mean maximum dose of 1 cc of duodenum (*D_1cc_*) was significantly higher for patients with grade ≥2 ulceration or stenosis (37.4 Gy vs. 25.3 Gy). However, patients receiving a dosage lower than 25.3 Gy only experienced grade 0 or 1 duodenal toxicity (42). Bae et al. also indicated that *D_0.03cc_* can be a valuable predictor of gastroduodenal toxicity, as the *D_0.03cc_* of 35 Gy and 38 Gy were respectively associated with a 5% and 10% probability of developing severe gastroduodenal toxicity (43). Thus, we used *D_0.03cc_* and *D_5cc_* to evaluate the safety of the duodenum, colon, stomach, and small bowel according to RTOG1112 and TG101 recommendations. The *D_0.03cc_* or *D_5cc_* of each organ met the criteria. Compared with HA1, the *D_0.03cc_* and *D_5cc_* of the gastrointestinal organs decreased in HA2. Meanwhile, only the *D_0.03cc_* and *D_5cc_* of the stomach continued to reduce in HA3. These results indicate that gastrointestinal OARs can be reduced by increasing the number of partial arcs, and the effect decreases by using three arcs.

Adding several arcs improved and helped spare the OARs. The stomach, kidneys, and bowels were the most significant ones. Normal liver was also protected by decreasing maximum dose in *D_700cc_* and *D_500cc_*, respectively.

However, it is important to acknowledge certain limitations in our study. Firstly, this was a retrospective analysis, potentially introducing selection bias. Secondly, our investigation was confined to a single-center setting, relying on a limited patient pool. The relatively large tumor sizes within our cohort may restrict the generalizability of our findings to specific patient groups. Lastly, the optimized plans have yet to be implemented for patient treatment. Assessing the clinical outcomes of patients treated with these optimized plans is essential for verifying their organ-sparing benefits. Moreover, comparing our proposed approach with alternative methods may hold promise for future research endeavors.

## 5 Conclusion

In the present study, we used an ASP to generate SBRT plans for oligo liver tumors. The process was more objective and had less dependence on physicians’ skills or preferences, which can help rule out manual bias. We comprehensively compared multiple-arc plans by using ASP. Using more arcs improved conformity but sacrificed planning homogeneity. In addition, increasing half-arcs improved dose distribution and dose fall-off setting. A sharper dose of fall-off planning showed prominent benefits in protecting OARs. However, the advantages were mostly found in two or three half-arc plans in the present study. Only the liver, kidney, and bowel were protected in the four-arc plan. All in all, using ASP may improve the consistency of the liver SBRT plan, and using three to four half arcs may improve plan conformity with better protection of surrounding OARs. On the other hand, the beam-on time was prolonged. Considering both treatment quality and efficiency, a three-arc plan is suitable for clinical application.

## Data availability statement

The raw data supporting the conclusions of this article will be made available by the authors, without undue reservation.

## Ethics statement

The studies involving humans were approved by the Clinical Research Committee of the study institute. The studies were conducted in accordance with local legislation and institutional requirements. The ethics committee/institutional review board waived the requirement of written informed consent for participation from the participants or the participants’ legal guardians/next of kin because this is a retrospective study without any intervention during patients’ previous treatment.

## Author contributions

SZ: Conceptualization, Data curation, Formal Analysis, Investigation, Methodology, Validation, Writing – original draft. WZ: Data curation, Formal Analysis, Writing – original draft. NZ: Investigation, Writing – original draft, Data curation, Methodology. JY: Data curation, Formal Analysis, Writing – original draft. MX: Investigation, Writing – review and editing. WL: Funding acquisition, Validation, Writing – review and editing. NC: Supervision, Validation, Writing – review and editing. JX: Conceptualization, Funding acquisition, Supervision, Validation, Writing – review and editing.
