# Supplementary material for: Dosimetric comparison in sparing normal tissue dosage by using auto-SBRT planning in oligo liver tumors
Source: Front Oncol. 2023 Nov 9;13:1273042. doi: 10.3389/fonc.2023.1273042 (PMC10665725; doi:10.3389/fonc.2023.1273042)
Supplement: Supplementary file 1 [file Table_1.docx]

Supplement Table 1. Summary of beam-on-time for different plans

|  | **HA1** | **HA2** | **HA3** | **HA4** | **P** |
| --- | --- | --- | --- | --- | --- |
|  | **Median (25%-75%)** | **Median (25%-75%)** | **Median (25%-75%)** | **Median (25%-75%)** |  |
| Beam-on-time (s) | 109.00(105.00, 111.50) | 128.00(124.00, 131.00) | 146.00(144.00, 149.00) | 177.00(176.00, 179.00) | <0.001 |
