# Supplementary material for: Dosimetric comparison in sparing normal tissue dosage by using auto-SBRT planning in oligo liver tumors
Source: Front Oncol. 2023 Nov 9;13:1273042. doi: 10.3389/fonc.2023.1273042 (PMC10665725; doi:10.3389/fonc.2023.1273042)
Supplement: Supplementary file 2 [file Table_2.docx]

**Supplement Table 2 Percentage difference in beam-on-time in different plans**

|  | **HA2 versus. HA1** | | **HA3 versus. HA2** | | **HA4 versus. HA3** |  |
| --- | --- | --- | --- | --- | --- | --- |
|  | **Median (25%-75%)** | ***P*** | **Median (25%-75%)** | ***p*** | **Median (25%-75%)** | ***p*** |
| Beam-on-time | -15.45% (-16.33%, -14.39%) | <0.001 | -12.77% (-13.94%, -11.42%) | <0.001 | -17.61% (-18.31%, -16.57%) | <0.001 |

Note: Percentage differences were calculated as (B − A)/A (A vs.B)
